# Necrotizing Fasciitis Following Herpes Zoster Ophthalmicus in an Immunocompromised Patient

**DOI:** 10.1155/2019/4534153

**Published:** 2019-01-20

**Authors:** Grazia Maria Cozzupoli, Daniele Gui, Valerio Cozza, Claudio Lodoli, Mariano Alberto Pennisi, Aldo Caporossi, Benedetto Falsini

**Affiliations:** Fondazione Policlinico Universitario A. Gemelli, Università Cattolica del S. Cuore, Rome, Italy

## Abstract

Necrotizing fasciitis (NF) is a rare infection that spreads rapidly along the subcutaneous soft tissue planes. NF rarely involves the periorbital region due to the excellent blood supply of this region. We report a case of periorbital necrotising fasciitis following herpes zoster (HZ) in an immunocompromised 70-year-old patient with a dramatically rapid evolution into septic shock. In our patient, the surprisingly rapid spread of the bacterial superinfection led the periorbital cellulitis to turn into frank NF within 2 hours, with an overwhelming evolution. Despite the prompt start of a systemic antibiotic therapy and the immediate surgical intervention, the patient had a septic shock; she was treated in ITU for 31 days and then discharged to a medical ward and eventually died for a mix of complications of the medical treatment and comorbidities. This case is unique because any documented cases of periorbital NF triggered by HZ had never led to a septic shock and death. Ophthalmologists should be aware that even common skin lesions caused by shingles can determine a dramatic clinical picture, in presence of predisposing factors.

## 1. Introduction

Group A *β*-haemolytic streptococcus is one of the most common human pathogens. It may cause life-threatening infections with three overlapping clinical presentations: toxic shock syndrome, necrotizing fasciitis (NF), and bacteraemia with no identifiable focus [1].

NF is a rare infection that spreads rapidly along the subcutaneous soft tissue planes. Initially presenting as cellulitis, the infection becomes widespread by bacterial dissection along superficial and deep fasciae [2]. NF presents with pale red, tense, swollen skin and severe pain. Within 1 to 2 days, the skin becomes cyanotic and blue–grey, with irregular erythematous borders [1, 3]. Frank cutaneous gangrene develops within 4 to 5 days, and the skin sloughs due to underlying suppuration by 8 to 10 days [1, 3]. The overwhelming bacterial load leads to marked systemic symptoms that may include shock and organ failure [2]. Early diagnosis and prompt treatment are crucial for the successful management of NF [2].

NF usually involves the extremities, abdominal wall, and groin and rarely involves the head, neck, and periorbital region [4].

We report a case of periorbital NF following herpes zoster (HZ) in an immunosuppressed adult patient with a dramatically rapid evolution into septic shock.

## 2. Case Presentation

A 70-year-old woman affected by Waldenström's Macroglobulinemia, under immunosuppressive therapy with melphalan, was admitted to the Emergency Department of Policlinico Universitario A. Gemelli for severe infection of the facial skin in the periorbital region of left eye. The patient had a medical history of recurrent episodes of herpetic keratitis in the left eye associated with periocular vesicles and erythema due to HZ. Consequently, the patient underwent a deep anterior lamellar keratoplasty, on February 2014, and a penetrating keratoplasty, on June 2016. Since the first surgery, the patient had been under prophylactic antiviral therapy with acyclovir. Furthermore, on January 2010, the patient underwent a right dacryocystorhinostomy.

The patient presented to the emergency room having developed periocular blistering, swelling, pain in the same left dermatome of the trigeminal nerve interested in the previous HZ episodes, and also fever in the past 2 days. A diagnosis of shingles was made, and the patient was subsequently prescribed topical and intravenous acyclovir and then discharged.

After 24 hours, the patient represented with worsening of the clinical picture. There were tense periorbital oedema, pain, and erythema spreading to the surrounding areas. The patient was persistently febrile (T≥38.7°C), tachycardic (HR≥105 bpm), and hypotensive (BP≤100/60 mmHg) requiring fluid resuscitation and inotropic support.

A provisional diagnosis of HZ ophthalmicus with secondary bacterial periorbital cellulitis was made. Intravenous piperacillin-tazobactam, clindamycin, linezolid, and acyclovir were initiated.

**Figure 1:**
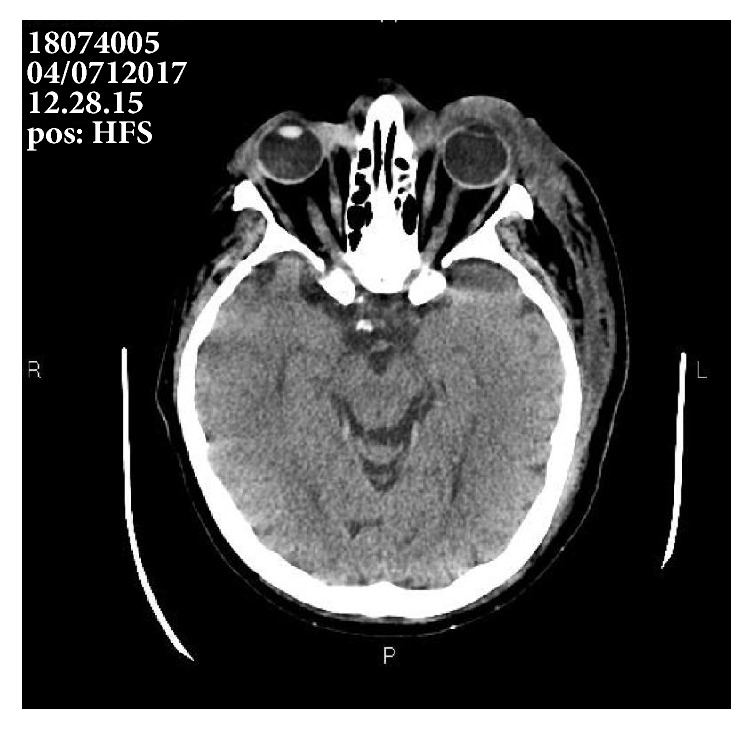
Computed tomography showing soft tissue swelling.

Non-contrast-enhanced and Iopromide-enhanced cranial computed tomography was urgently performed, showing soft tissue swelling in left periorbital, frontal, temporal, and zygomatic region and at parietal level bilaterally, up to the vertex. The swelling continued caudally to the subcutaneous tissue of the left cheek, reaching the submental and neck region. No evidence of sinus involvement was found (Figure 1).

Despite the adequate fluid administration and the antibiotic and antiviral therapy, in 2 hours the status of the patient evolved into severe hemodynamic instability (HR of 125 bpm, sinus rhythm, BP< 90/40 mmHg) with visible increase in the soft tissue oedema, persistent metabolic acidosis, high blood lactate levels, malaise, and confusion.

The clinical picture of the patient was consistent with the diagnosis of septic shock secondary to periorbital necrotizing fasciitis.

The patient was immediately transferred to the intensive care unit for cardiovascular monitoring. Orotracheal intubation was performed, high-dose adrenaline infusion started, piperacillin/tazobactam discontinued, and imipenem/cilastatin 1 g intravenously every 6 hours added.

**Figure 2:**
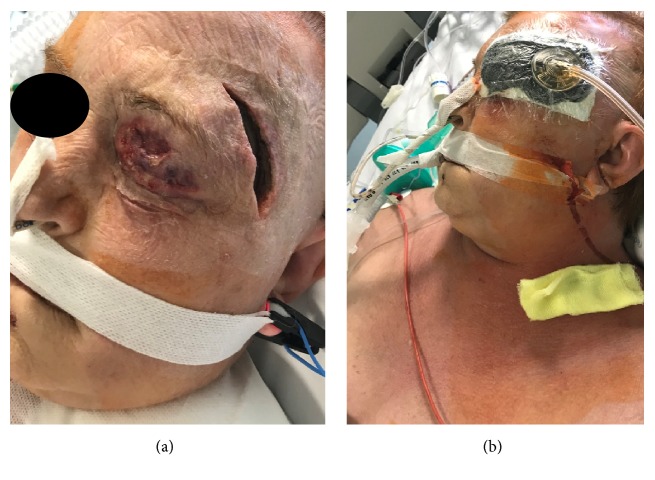
(a) The left frontotemporal surgical incision. (b) NPWT dressings positioned at this level.

The patient was then referred to the general surgery department and was taken for prompt debridement and fasciotomy for necrotising fasciitis. Two surgical incisions were performed at left frontotemporal and supraclavicular region proceeding with the fasciotomy of temporal and platysma muscle. At the time of the surgery, no purulent discharge was noticed at any levels. All tissue biopsies were reviewed by a consultant pathologist. The patient underwent further surgical debridement after 18 hours. Left upper eyelid showed substantial necrosis of the skin, pretarsal orbicularis muscle, orbital septa, and fat pads. The temporal muscle fascia was also involved by the necrosis and a purulent discharge from the subcutaneous soft tissue at the surgical incisions was observed. Diffuse induration and erythema persisted at left face, neck, and supraclavicular region. Drainage and debridement of the surgical sites were completed and a Negative Pressure Wound Therapy (NPWT) started (Figures 2(a) and 2(b)). The supraclavicular wound was treated with NPWT for 3 days and then substituted by conventional dressings. The frontotemporal wound was treated with NPWT for 10 days (with wound dressing change every 48-72 hrs) and then conventional dressings.

Samples taken from the infected tissues showed group A haemolytic* Streptococcus* pyogenes infection with histopathological features suggestive of necrotising fasciitis, in keeping with the clinical picture.

On day 7 after surgery, the oedema and erythema of left frontotemporal and supraclavicular region and neck were healed up. Throughout her admission, she received regular ophthalmology review. Ocular bulb integrity and corneal graft remained preserved at all times; an eschar formed on the upper left lid with clear reduction of the periorbital swelling. She was prescribed a tetracycline unguent.

On day 13 after surgery, the patient was diagnosed with postsepsis critical illness myopathy and neuropathy [5], confirmed by electromyography of the deltoid and biceps brachii muscles.

On day 28 after surgery, the limbs resulted in severe hypoperfusion and ischemia, thus into wet gangrene, due to the high-dose adrenaline therapy and, possibly, the underlying Waldenström's Macroglobulinemia, responsible for vasculitis and hyperviscosity syndrome. Contrast-enhanced MRI of the limbs showed a gangrene demarcation line more proximal than clinically expected. Necrosis and ischemic damage extended up to all the limbs muscles, predicting a dismal prognosis in the short term. Given the extent of gangrenous area, the only radical intervention seemed to be the hindquarter and forequarter amputation. However, the team of orthopaedic and general surgeons judged this demolitive procedure disproportioned and contraindicated it. The relatives were informed about the critical conditions of the patient. A bioethics consultant was called in to assess the case. In consideration of the irreversible evolution of the clinical picture, the consultant confirmed the unfavourable risk-benefits ratio of the aforementioned procedure.

After 31 days spent in the intensive care unit, the patient was assigned to palliative domiciliary care and died after a total of 61 days from surgery.

## 3. Discussion

A review [6] of 163 patients with NF showed that only 10% involved the head and neck. As described in an observational retrospective case series conducted in 2006 [7], the disease even more rarely involves the eyelids, with only 58 well-documented case reports. In these case reports, the risk factors [8, 9] for developing NF to the eyelids included alcoholism (26%), diabetes mellitus (10%), rheumatologic disease (7%), systemic malignancy (1%), and systemic corticosteroid use (1%). Most patients (52%), however, were healthy, without any predisposing conditions [7].

NF may involve both the eyelids because the subcutaneous tissue over the nose has little resistance to the spread of infection. Of reported cases, 45% had bilateral involvement and 55% had unilateral involvement [7].

NF has a different clinical course in the eyelids than elsewhere in the body due to the very thin eyelid skin with a rich blood supply [2, 4, 7]. The eyelids have little subcutaneous fat, so skin changes and necrosis following infection are visible at an early stage and patients present for medical assistance sooner [10]. Furthermore, the orbicularis muscle acts as a barrier for deep full-thickness spread. Thicker dermis at the eyebrows and malar folds and the dermal adherence at the inferior and lateral margins prevent further extension onto the cheek and forehead [10, 11]. The lid margins are often spared because of the rich blood supply from the marginal arterial arcade [10, 12]. It is because of these anatomic features and early recognition that periorbital NF has a lower mortality rate than truncal or limb NF [4, 10].

Four different case reports [10, 13–15] describe an uncommon association between herpes zoster infection, acting as a trigger, and the development of necrotising fasciitis. In two of the cases mentioned above, the trunk was affected: one patient was a 26-year-old woman without any predisposing conditions [13]; another one was an immunosuppressed 76-year-old woman [14]. In the remaining two cases, the periorbital region was affected: the upper left eyelid of an immunodepressed 53-year-old man [15] (case 1 in Table 1); both eyelids of an immunocompetent 63-year-old woman [10] (case 2 in Table 1).

The vesiculopapular rash arising from herpes zoster may have provided the entry portal for Streptococcal infection in all the cases listed above [10]. Of the two cases involving the periocular region, none resulted in permanent visual impairment or in the development of septic shock.

We compare this patient with two previous cases of periorbital NF following herpes zoster ophthalmic lesions just published (Table 1).

Our patient was immunosuppressed, inasmuch as she was affected by Waldenström's Macroglobulinemia under chronic therapy with melphalan. Notably, the patient had a history of recurrent episodes of HZ ophthalmicus in the same left dermatome of the trigeminal nerve. Interestingly, the incidence of recurrent HZ is increased in patients with malignancy, immunological diseases, human immunodeficiency virus (HIV) infection, diabetes mellitus, and treatment with tumor necrosis factor-*α* inhibitors [16]. Moreover, Shiraki et al. observed recurrences of HZ in the same dermatome in 16.3% of their patients, and more frequently the recurrences occurred in the left side [16]. The reason for the left-side prevalence is not clear; however, similar observations on right- versus left-side asymmetry have been reported in muscle involvements, such as poliomyelitis of the upper limb, diaphragm paralysis, and neonatal hip instability [16]. Possibly, the patient's status of immunodeficiency and immunosuppression could be involved in allowing for a recurrence of the zoster infection in the same dermatome.

Also, this condition certainly acted as a predisposing factor for the dramatic evolution of the clinical picture. In our patient, the surprisingly rapid spread of the bacterial superinfection led the periorbital cellulitis to turn into frank NF within 2 hours, with an overwhelming evolution, despite the use of NPWT. Many publications outline the use of NPWT dressings, mainly Vacuum Assisted Closure (V.A.C.®, Acelity and KCI, San Antonio, TX) in the treatment of sternal, sacral, upper and lower extremity, perineal, and abdominal wounds but fewer describe its use in the head and neck region [17]. In 2013, Reiter and Harreus [18] reported on 23 patients treated with VAC therapy in the head and neck, of which six had NF. Byrnside et al. [19] described the use of VAC for necrotizing fasciitis of the face, in which skin was grafted at day 14 to the cheek and temple wounds after sufficient wound bed preparation with the VAC device [17].

This case is unique because any documented cases of periorbital NF triggered by HZ had never resulted in such a massive systemic involvement. Ophthalmologists should be aware that even common lesions caused by shingles can determine a dramatic clinical picture, in presence of predisposing factors.

## Figures and Tables

**Table 1:** Comparison between three cases of periorbital NF following cutaneous herpes zoster.

	Case 1	Case 2	Case 3 (present)
Age (yrs)/Gender	53/M	63/F	70/F

Immune System	Immunodepressed	Immunocompetent	Immunosuppressed

History of ocular diseases	No	No	2 previous keratoplasties for herpetic keratitis

General risk factors	Alcohol abuse	Discoid Lupus Erythematosus	Waldenström's Macroglobulinemia

Local risk factors	Periocular shingles lesions, recent eyelid trauma	Periocular shingles lesions	Periocular shingles lesions

Periorbital skin lesions localization	Right lower eyelid	Upper eyelids	Left upper eyelid

Wound Culture Results	Group A *β*-haemolytic streptococcus, staphylococcus aureus	Group A *β*-haemolytic streptococcus	Group A *β*-haemolytic streptococcus

History of Septic Shock	No	No	Yes

Time btw. diagnosis of periorbital cellulitis and development of NF	8 days	18 hours	2 hours

Systemic Antibiotic Treatment	Co-amoxiclav and acyclovir	Clindamycin, ciprofloxacin and acyclovir	Imipenem/cilastatin, clindamycin, linezolid and acyclovir

Surgical Debridement	Debridement, reconstructive full-thickness skin grafting to the lower lid	Debridement, full-thickness skin grafting to the upper lids	Debridement, fasciotomy

Outcome	Survived; donor and graft sites healthy.	Survived; donor and graft sites healthy.	Died.
